# Prof W. H. Eames, D. D. S.

**Published:** 1894-04

**Authors:** A. W. Harlan, Truman W. Brophy, Edgar D. Swain


PROF. W. H. EAMES, D. D. S.—Resolutions on the death of Professor W. H. Eames, of St. Louis, were adopted at the regular meeting of the Chicago Dental Society, April 3rd, 1894.

Whereas, it has come to the knowledge of the members of the Chicago Dental Society that the dental profession is called to mourn the loss of one of its most distinguished and honored members.

Therefore be it resolved that in the decease of Prof. W. H. Eames, the world has lost one of its foremost educators, his family a devoted husband and father, and the profession a sincere, earnest and useful devotee. It is fitting that this society should pay its tribute of respect to one who has so long and honorably filled positions before the world and we mourn his death as a personal loss to each and all of us.

Be it further resolved that a copy of these lines be transmitted to the family of the deceased and a further copy be sent to the journals for publication.

A. W. HARLAN,
TRUMAN W. BROPHY,
EDGAR D. SWAIN.

